# Upscaling of Helmholtz Equation Originating in Transmission through Metallic Gratings in Metamaterials

**DOI:** 10.1155/2016/7436136

**Published:** 2016-09-21

**Authors:** Hari Shankar Mahato

**Affiliations:** College of Engineering, University of Georgia, Athens, GA 30602, USA

## Abstract

We investigate the transmission properties of a metallic layer with narrow slits. We consider (time-harmonic) Maxwell's equations in the *H*-parallel case with a fixed incident wavelength. We denote *η* > 0 as the typical size of the complex structure and obtain the effective equations by letting *η* → 0. For metallic permittivities with negative real part, plasmonic waves can be excited on the surfaces of the slits. For the waves to be in resonance with the height of the metallic layer, the corresponding results can be perfect transmission through the layer.

## 1. Introduction

Negative refraction of electromagnetic waves in* metamaterials* has become of major interest in recent years, compare [1, 2], especially to construct small scale optical devices for technical applications in the fields of micro- and nanooptics. Metamaterials are the materials that are not found in nature; instead they are created by the composition of several metals or plastics or both. Due to their precise shape, size, geometry, and arrangement of metals, these metamaterials are capable of influencing the* electromagnetic waves* by absorbing, bending, or refracting. To create the metamaterials, the composite materials are arranged in repeated (periodic) fashion with periodicity scales smaller than the wavelength of waves. Negative index metamaterial or negative index material (NIM) is a metamaterial where the refractive index (in optics theory, the refractive index of a material is a dimensionless number which describes how light propagates through that medium and is defined as the ratio *c*/*υ*, where *c* is the speed of light in vacuum and *υ* is the phase velocity of light in the medium) has a negative value over some frequency range when an electromagnetic wave passes through it. Negative index materials are extensively studied in the fields on optics, electromagnetics, microwave engineering, material sciences, semiconductor engineering, and several others.

**Figure 1:**
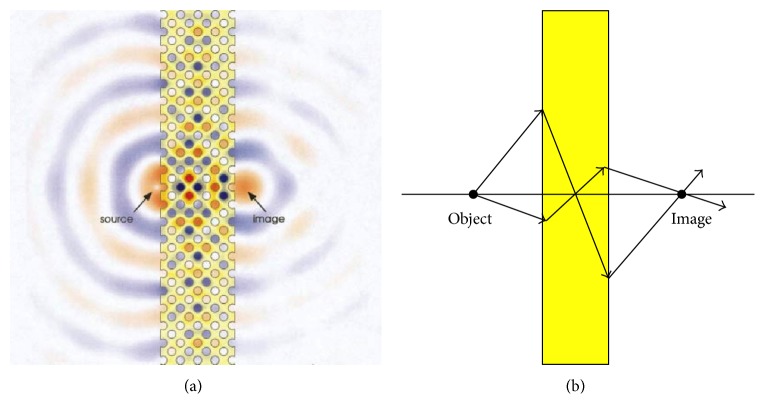
The light wave is originating from the left side and is getting refracted through the metallic plate and its image is on the right (this image is taken from [2]).

In this work, we study the phenomena of light wave passing through the subwavelength metallic structure; that is, we investigate the high transmission of light wave through a metamaterial with thin holes inside it. We consider a thin metallic structure (inside a medium) with holes smaller than the wavelength of incident photon which shows the high transmission of light waves through this metallic structure. This high transmission contradicts the classical aperture theory and shows an important feature of metamaterials. To demonstrate the geometry assumed in this work, let us consider Figure 1 where the light wave emerging from a source (l.h.s of the figure) is passing through a metamaterial with negative refractive index and its image is given on the r.h.s. (cf. this figure to that of [3]).

The holes inside the metallic layer are periodically distributed with period *η* > 0 smaller than the wavelength *λ* of incident light wave. This layer can be considered as a heterogeneous or perforated media and our goal is to give a physically consistent approach to transmission properties of heterogeneous media using the techniques from homogenization theory and applied analysis. We obtain an effective (upscaled) scaterring problem where the metallic layer with holes is replaced by a homogenized structure with effective permittivity *ε*_eff_ and permeability *μ*_eff_. We also obtain the tranmission coefficient *T* in terms of incident wave number *k* and incident angle *θ*. We will see that, for lossless materials with (real) negative permittivity *ε*_*η*_, perfect transmission *T* = 1 can be obtained for every *θ* and suitable value for *k*. In the recent times, several significant investigations for metamaterials have been done. In [4] the connection between the high transmission and the excitation of surface plasmon polaritons has been established. The photonic band structure of the surface plasmons is evaluated numerically, In [5] the authors have calculated the transmission coefficients for the lamellar gratings, while the effect of surface plasmons on the upper and lower boundary of the layer is investigated in [6]. In [7], the effect of finite conductivity is studied. In [8], the relation between the high transmission effect and the negative index material is obtained with a fishnet like structure. A homogenization method is proposed in [9] where the author accentuates the connection between the skin depth of evanescent modes in the metallaic structure and the period of the gratings. Some results in this direction can be found in [10–13].

*Two-scale convergence* has proven to be a very efficient tool in homogenization theory while dealing with the problems where the underlying medium is heterogenous. The concept of two-scale convergence is first introduced by Nguetseng in [14]. This convergence criterion and the results related to it have been used extensively in the homogenization of partial differential equations; see Allaire [15, 16], Cioranescu and Donato [17], and Mahato and Böhm [18]. In this work too, we have used the two-scale convergence of an oscillating sequence and its gradient; see Section 1.4. At this point we would like to point out that the geometry of metallic structure in this work is generalized compared to that considered in [3]. In [3] due to the rectangular shapes of the metallic gratings, the coefficients of the effective system were determined by the help of a scalar, one-dimensional shape function *ψ* : *ℝ* → *ℂ* given by the hyperbolic functions; however in our work where the considered geometry is more realistic, such nice representation is not possible. To deal with this problem in this work an eigenvalue approach has been proposed and this eigenvalue problem in the unit cell *Y* helps us to determine the effective parameters of the problem.

Although this paper can be compared with [3] in some way, the major difference in this work is that our limit function ${\vec{j}}_{0}$ of ${\vec{j}}_{\eta }=a_{\eta }\nabla u_{\eta }$ (see Section 1.5 for details) and the limit function ${\vec{j}}_{0}$ in [3] are totally different. In [3], the authors worked with a rectangular metallic subpart Σ of type (−*γ*, *γ*)×(−1/2, 1/2) and therefore by defining a suitable test function, they have shown that the first component of *j*_0_ vanishes and they obtain *j*_0_ = (0, *α* ^−1^∂_*x*_2__ *U*)  for  *x* ∈ *R*  and  *y* ∈ *Y*∖Σ. This is clearly not the case in this paper as the metallic subpart Σ is chosen to be sinusoidal along *y*_2_-axis due to the geometry of the metallic structure given by Figure 1. This will lead to nonvanishing componenets of *j*_0_ and we end up having a different *j*_0_ compared to that in [3] and hence, we will obtain a different upscaled equation. Also no explicit representation of ${\vec{j}}_{0}$ can be obtained due to the geometry of Σ.

In Sections 1.1, 1.2, and 1.3, we will outline the model in detail. In Section 2, we gather some mathematical tools required to do the analysis and we state our main results. In Sections 3 and 4 we will prove the main results.

### 1.1. Model

We investigate the time-harmonic solutions of a Maxwell equations with a fixed wave number *k* and the corresponding wave length *λ* = 2*π*/*k*. Let the metallic structure remain unchanged towards *x*_3_-direction and the metallic field, denoted by $\vec{H}$, is parellel to *x*_3_; that is, $\vec{H}=(0,0,u)$, where *u* : *ℝ* ^2^ → *ℝ*.

The heterogenoeus domain *Ω* has a metallic structure of finite length and finite height in *ℝ* ^2^, and the slits (vacuum) are repeated periodically with a small period *η* > 0, compare Figure 1. The period *η* is assumed to be infinitesimally small with respect to the wavelength *λ*. The relative permittivity of the metal is denoted by *ε*. Since the permittivity of conductors has large absolute values, we assume that it depends on *η* and consider *ε* = *ε*_*η*_. We obtain nontrivial effects due to plasmonic resonance for |*ε*_*η*_| ∝ *η* ^−2^, compare [3, 13]. If Σ_*η*_ denotes the matallic part in *Ω*, we set

(1) $$\begin{array}{c} \varepsilon_{\eta }\left(\;x\;\right)≔\left\{\begin{array}{cc} 1 & for\;\;x\notin \Sigma_{\eta },\\ \frac{\varepsilon_{r}}{\eta^{2}} & for\;\;x\in \Sigma_{\eta }, \end{array}\right. \end{array}$$

where *ε*_*r*_ ∈ *ℂ*. Due to ohmic losses inside the metal, Im⁡(*ε*_*η*_) is always assumed to be positive in a physical system which means we always take Im⁡(*ε*_*r*_) ≥ 0 and *ε*_*r*_ ≠ 0. A material is called a* lossless material *if Im⁡(*ε*_*r*_) = 0. Our particular interest is to study a lossless material with negative relative permittivity; that is, Im⁡(*ε*_*r*_) = 0 and Re⁡(*ε*_*r*_) < 0. For such *ε*_*r*_ transverse evanescent modes will be generated in the metal. Since Re⁡(*ε*_*r*_) < 0 and Im(*ε*_*r*_) = 0, then from (14) we have $u_{\eta }(x_{1},x_{2})=(A_{1}e^{\bar{k}x_{1}}+A_{2}e^{-\bar{k}x_{1}})(B_{1}x_{2}+B_{2})$; that is, we can obtain wave like solutions and waves cannot penetrate the metallic grating. These evanescent modes can penetrate only in a region which is given by the skin depth of order *η*. The evanascent mode is related to a surface plasmon solution (in this case a solution which is nonvanishing in the grating but which has exponential decay in the metal). The main aspect of the current work is to generalize the geometric structure of the metallic slab inside *Ω* given in [3].

### 1.2. Geometry

Let *η* > 0 be a small scale parameter and *Ω* be the domain under investigation which is bounded in *ℝ* ^2^. Let *Y*≔(−1/2, 1/2)×(−1/2, 1/2) be the representative unit cell in *ℝ* ^2^ and Σ be an open set in *Y* such that $\bar{\Sigma }\subset Y$ and *Y*≔Σ ∪ (*Y*∖Σ). Let us choose Σ in such a way that it follows a* sinusoidal* profile along *y*_2_-axis; that is,

(2) Σ≔y1,y2:y2≤12,  y1∈Sγ,  Sγ≔−3γ4+γ4cos2πy2,3γ4+γ4cos2πy2,  γ∈0,12.

Keeping physics of the problem in mind, Σ denotes the mettalic part which lies between the two columns of holes in the metallic structure of type introduced in Figure 1.

**Figure 2:**
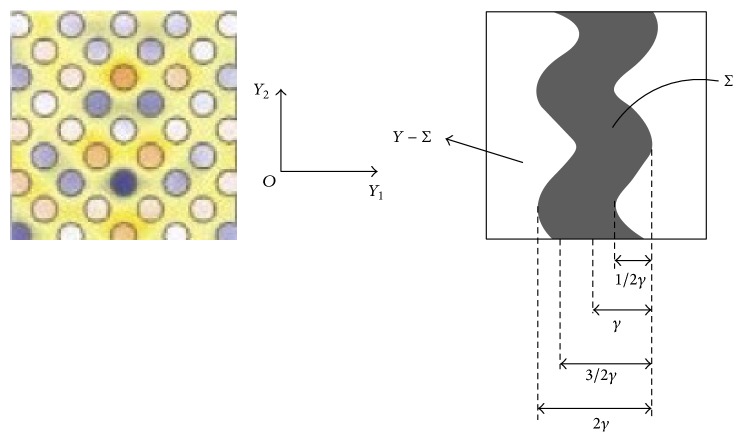
The representative cell *Y* ⊂ *ℝ* ^2^ (cf. this figure with figure  1 in [3]).

**Figure 3:**
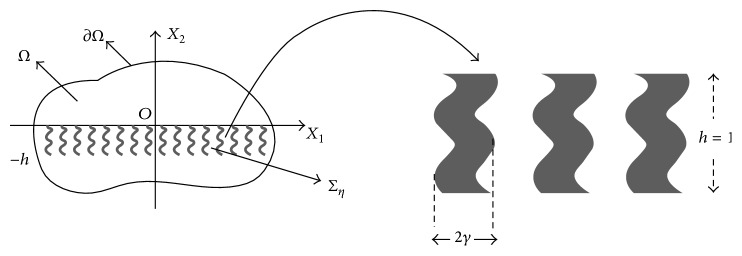
The metallic structure Σ_*η*_ inside the domain *Ω*.

The relative aperture volume *α* = 1 − 3*γ*/2 ∈ (1/4, 1) and relative metal volume is (3/2)*γ*, where *γ* ∈ (0, 1/2). We define 2*N*_*η*_ + 1≔2*l*/2*ηγ*. We assume that the compact rectangle $\bar{R}$ contains (2*N*_*η*_ + 1) number of small rectangles of type (*nη* − *ηγ*, *nη* + *ηγ*) × (−*h*, 0), that is, of width 2*γη* and height *h*, which include the *η*-scaled versions of the metallic part Σ (cf. Figures 2 and 3), where each Σ is of width (3/2)*γη* and height *h*. The collection of these small *η*-scaled versions of the metallic part is the metallic domain Σ_*η*_ and assume that the two-dimensional heterogeneous metallic structure introduced in Figure 1, denoted by Σ_*η*_ and parellel to *x*_1_-axis, is contained in the closure of the set *R*≔(−*l*, *l*)×(−*h*, 0) ⊂ *Ω* with $\bar{R}\subset \Omega$; that is;

(3) $$\begin{array}{c} \Sigma_{\eta }\subset \overset{N_{\eta }}{\underset{n=-N_{\eta }}{⋃}}\left(\;n\eta -\eta \gamma ,n\eta +\eta \gamma \;\right)\times \left(\;-h,0\;\right)\subset \bar{R}\subset \subset \Omega , \end{array}$$

see Figure 3.

As *η* → 0, *N*_*η*_ → *∞*. Due to nondimensionalization, we are, however, only interested in *h* = 1.

### 1.3. Function Spaces

Let *θ* ∈ [0,1] and 1 ≤ *r*, *s* ≤ *∞* be such that 1/*r* + 1/*s* = 1. Assume that Ξ ∈ {*Ω*, Σ_*η*_} and *l* ∈ *ℕ*_0_; then as usual *L* ^*r*^(Ξ) and *H* ^*l*,*r*^(Ξ) denote the Lebesgue and Sobolev spaces with their usual norms and they are denoted by ‖·‖_*r*_ and ‖·‖_*l*,*r*_. For the sake of clarity if *ϕ* ∈ *L* ^*r*^(Ξ), then

(4) $$\begin{array}{c} {\left\|\;\phi \;\right\|}_{L^{r}\left(\Xi \right)}=\left\{\begin{array}{cc} {\left[\int_{\Xi }^{}{\left|\phi \left(x\right)\right|}^{r}dx\right]}^{1/p} & for\;\;1\leq r<\infty ,\\ \underset{x\in \Xi }{\mathrm{ess}\sup}\left|\phi \left(x\right)\right| & for\;\;r=\infty , \end{array}\right. \end{array}$$

and if *ϕ* ∈ *H* ^*l*,*r*^(Ξ), then

(5) ϕl,rϕHl,rΞ≔∑α≤l∫ΞDαϕrdx1/rfor  1≤r<∞,∑α≤less⁡supx∈Ξ⁡Dαϕxfor  r=∞,

where *α* = (*α*_1_, *α*_2_,…, *α*_*n*_) ∈ *ℕ* ^*n*^ is a multi-index, |*α* | = *α*_1_ + *α*_2_ + ⋯+*α*_*n*_, and *D* ^*α*^ = ∂^|*α*|^/∂_*x*_1__^*α*_1_^∂_*x*_2__^*α*_2_^ ⋯ ∂_*x*_*n*__^*α*_*n*_^. Similarly, $C^{\theta }(\bar{\Xi })$, (·, ·)_*θ*,*r*_, and [·, ·]_*θ*_ are the Hölder, real, and complex interpolation spaces, respectively, endowed with their standard norms; for definition confer [19, 20]. *C*_#_^*α*^(*Y*) denotes the set of all* Y-periodic α-times* continuously differentiable functions in *y* for *α* ∈ *ℕ*. In particular, *C*_#_(*Y*) is the space of all the* Y-periodic* continuous function in *y*. The *C* ^*∞*^-spaces are as usual equipped with their* maximum norm* whereas the space of all continuous functions *C*(Ξ) is furnished with* supremum norm*, compare in [19].

### 1.4. Two-Scale Convergence

**Definition 1 .**

A sequence of functions (*u* ^*η*^)_*η*>0_ in *L* ^*r*^((0, *T*) × *Ω*) is said to be two-scale convergent to a limit *u* ∈ *L* ^*r*^((0, *T*) × *Ω* × *Y*) if

(6) limη→0∫0T∫Ωuηt,xϕt,x,xηdx dt=∫0T∫Ω∫Yut,x,yϕt,x,ydx dt dy

for all *ϕ* ∈ *L* ^*s*^((0, *T*) × *Ω*; *C*_#_(*Y*)).

By $\overset{2}{⇀}$,$\;\overset{w}{⇀}$, and → we denote the two-scale, weak, and strong convergence of a sequence, respectively. Finally, *S* = (0, *T*) denotes the time interval.

**Lemma 2 (cf. [21]).**

For every bounded sequence (*u* ^*η*^)_*η*>0_ in *L* ^*r*^(*S* × *Ω*) there exists a subsequence (*u* ^*η*^)_*η*>0_ (still denoted by same symbol) and *u* ∈ *L* ^*r*^((0, *T*) × *Ω* × *Y*) such that $u^{\eta }\overset{2}{⇀}u$.

**Lemma 3 (cf. [21]).**

Let (*u* ^*η*^)_*η*>0_ be strongly convergent to *u* ∈ *L* ^*r*^((0, *T*) × *Ω*), and then $u^{\eta }\overset{2}{⇀}u_{1}$, where *u*_1_(*t*, *x*, *y*) = *u*(*t*, *x*).

**Lemma 4 (cf. [21]).**

Let (*u* ^*η*^)_*η*>0_ be a sequence in *L* ^*r*^((0, *T*); *H* ^1,*r*^(*Ω*)) such that $u^{\eta }\overset{w}{⇀}u$ in *L* ^*r*^((0, *T*); *H* ^1,*r*^(*Ω*)). Then $u^{\eta }\overset{2}{⇀}u$ and there exists a subsequence (*u* ^*η*^)_*η*>0_, still denoted by same symbol, and *u*_1_ ∈ *L* ^*r*^((0, *T*) × *Ω*; *H*_#_^1,*r*^(*Y*)) such that $\nabla_{x}u^{\eta }\overset{2}{⇀}\nabla_{x}u+\nabla_{y}u_{1}$.

**Lemma 5 .**

Let (*u* ^*η*^)_*η*>0_ be a bounded sequence of functions in *L* ^*r*^(*S* × *Ω*) such that *η*∇*u* ^*η*^ and *η* ^1/2^∇*u* ^*η*^ are bounded in *L* ^*r*^(*S* × *Ω*)^*n*^. Then there exists some functions *u*, *u*_1_ ∈ *L* ^*r*^(*S* × *Ω*; *H*_#_^1,*r*^(*Y*)) such that $u^{\eta }\overset{2}{⇀}u$, $\eta \nabla u^{\eta }\overset{2}{⇀}\nabla_{y}u$, and $\eta^{1/2}\nabla u^{\eta }\overset{2}{⇀}\nabla_{y}u_{1}$.

**Proof**

(i) Since *u* ^*η*^ and *η*∇*u* ^*η*^ are bounded sequence of functions in *L* ^*r*^(*S* × *Ω*) and *L* ^*r*^(*S*; *L* ^*r*^(*Ω*))^*n*^, respectively, then there exists *u* ∈ *L* ^*r*^(*S* × *Ω* × *Y*) and *U* ∈ *L* ^*r*^(*S* × *Ω* × *Y*)^*n*^ such that $u^{\eta }\overset{2}{⇀}u$ and $\eta \nabla u^{\eta }\overset{2}{⇀}U$ as *η* → 0. This means that, for the sequence *η*∇*u* ^*η*^, we have

(7) $$\begin{array}{c} \eta \overset{}{\underset{S\times \Omega }{\int }}\nabla u^{\eta }\cdot \vec{\phi }\left(\;x,\frac{x}{\eta }\;\right)dx\;dt\;\overset{\eta \to 0}{\to }\\ \overset{}{\underset{S\times \Omega \times Y}{\int }}U\left(\;x,y\;\right)\cdot \vec{\phi }\left(\;x,y\;\right)dx\;dy\;dt \end{array}$$

for all $\vec{\phi }\in C_{0}^{\infty }(S\times \Omega ;C_{\#}^{\infty }(Y){)}^{n}$. We integrate by parts the l.h.s, and which gives

(8) limη→0⁡ η∫S×Ω∇uηx·ϕ→x,xηdx dt=−limη→0⁡∫S×Ωuηx·η∇x·ϕx,xη+∇y·ϕx,xηdx dt=−∫S×Ω×Yux,y∇yϕx,ydx dy dt=∫S×Ω×Y∇yux,yϕx,ydx dy dt.

And it follows from (7) and (8) that *U*(*t*, *x*, *y*) = ∇_*y*_ *u*(*t*, *x*, *y*).

(ii) To prove the second part of the lemma, let us choose $\phi (t,x,x/\eta )=\phi_{1}(t,x)+\sqrt{\eta }\phi_{2}(t,x,x/\eta )$, where ${\vec{\phi_{1}}}_{}\in C_{0}^{\infty }(S\times \Omega {)}^{n}$ and ${\vec{\phi_{1}}}_{}\in C_{0}^{\infty }(S\times \Omega ;C_{\#}^{\infty }(Y){)}^{n}$. Note that the boundedness of *η* ^1/2^∇*u* ^*η*^ implies the boundedness of *η*∇*u* ^*η*^ in *L* ^*r*^(*S* × *Ω*) and hence, by part (i) there exists *u* ∈ *L* ^*r*^(*S* × *Ω*; *H*_#_^1,*r*^(*Y*)) such that $u^{\eta }\overset{2}{⇀}u$ and $\eta \nabla u^{\eta }\overset{2}{⇀}\nabla_{y}u$. Now let us assume that $\eta^{1/2}\nabla u^{\eta }\overset{2}{⇀}U$, and then by definition

(9) limη→0⁡ η1/2∫S×Ω∇uηϕ1t,x+ηϕ2t,x,xηdx dt=limη→0⁡∫S×Ωη1/2∇uηϕ1t,x+η∇uηϕ2t,x,xηdx dt=∫S×Ω×YUt,x,yϕ1t,x+∇yut,x,y·ϕ2t,x,ydx dy dt.

We integrate by parts the l.h.s.; then

(10) −limη→0⁡∫S×Ωη1/2uηx∇xϕ1t,x︸bounded+ηuηx∇xϕ2t,x,xη︸bounded+uηx∇yϕ2t,x,xηdx dt=−∫S×Ω×Yut,x,y∇yϕ2t,x,ydx dy dt.

We compare (9) and (10) which leads us to

(11) $$\begin{array}{c} \overset{}{\underset{S\times \Omega \times Y}{\int }}U\left(\;t,x,y\;\right)\phi_{1}\left(\;t,x\;\right)dx\;dy\;dt=0. \end{array}$$

Since *ϕ*_1_ is independent of *y* and ∇_*y*_ · *ϕ*_1_(*t*, *x*) = 0, from (11) it follows that *U* must be the gradient of some function *u*_1_ ∈ *L* ^*r*^(*S* × *Ω*; *H*_#_^1,*r*^(*Y*)) such that *U*(*t*, *x*, *y*) = ∇_*y*_ *u*_1_(*t*, *x*, *y*); that is, $\eta^{1/2}\nabla u^{\eta }\overset{2}{⇀}\nabla_{y}u_{1}(t,x,y)$. This completes the proof.

### 1.5. Mathematical Formulation and Statement of the Main Results

We study the Maxwell equations in a complex geometry with highly oscillating permittivities. By *η* we denote (i) the dimensionless positive scale parameter which represents the small length scale in the geometry Σ_*η*_ ⊂ *ℝ* ^2^ and (ii) the oscillations of large absolute values of the permittivity. We follow the standard nondimenionsalization techniques; for instance, see [3, 18, 22], and so forth and from here on all the quantities considered in this work are dimensionless unless stated otherwise. For the electric field ${\vec{E}}_{\eta }$ and magnetic field ${\vec{H}}_{\eta }$, the time-harmonic Maxwell equations are

(12) ∇×E→η=iωμ0H→η,∇×H→η=−iωεηε0E→η,

with fixed positive real constants *ω*, *μ*_0_, and *ε*_0_ denoting the frequency of the incident waves and the permeability and the permittivity of vacuum, respectively. We postulate that all the quantities are *x*_3_-independent and the polarized magnetic field is given by ${\vec{H}}_{\eta }=(0,0,u_{\eta })$, where *u*_*η*_ : *Ω* ⊂ *ℝ* ^2^ → *ℝ*. By orthogonal property of ${\vec{E}}_{\eta }$ and ${\vec{H}}_{\eta }$, we have ${\vec{E}}_{\eta }=(E_{x,\eta },E_{y,\eta },0)$. Then (12) reduce to

(13) −∂Ex,η∂y+∂Ey,η∂x=iωμ0uη,∂u∂y,−∂u∂x=−iωεηε0Ex,η,Ey,η.

By (13), a straightforward calculation yields

(14) $$\begin{array}{c} \nabla \cdot \left(\;\varepsilon_{\eta }^{-1}\nabla u_{\eta }\;\right)=-k^{2}u_{\eta }, \end{array}$$

where we have set *k* ^2^ = *ω* ^2^ *ε*_0_ *μ*_0_. We define the coefficient *a*_*η*_(*x*)≔*ε*_*η*_^−1^(*x*) which can have a negative real part and that it vanishes in the metal as *η* → 0. Thus we have the desired Helmholtz equation which we will study in this paper and is given below. We study solutions *u*_*η*_ ∈ *H*_loc_^1^(*Ω*) of

(15) $$\begin{array}{c} \nabla \cdot \left(\;a_{\eta }\nabla u_{\eta }\;\right)=-k^{2}u_{\eta }\;in\;\;\Omega , \end{array}$$

where the coefficient *a*_*η*_ is given by

(16) $$\begin{array}{c} a_{\eta }≔{\left(\;\varepsilon_{\eta }\;\right)}^{-1}=\left\{\begin{array}{cc} 1 & in\;\;\Omega \setminus \Sigma_{\eta },\\ \eta^{2}\varepsilon_{r}^{-1} & in\;\;\Sigma_{\eta }. \end{array}\right. \end{array}$$

The set Σ_*η*_ ⊂ ⊂*R* ⊂ *Ω* describes the complex geometry of the metallic inclusion in *Ω*; see Figure 3.

**Remark 6 (scattering problem).**

We will investigate the effective behavior of solutions of (15) in two different cases. In the first case we will study an arbitrary bounded sequence of solutions on a bounded domain *Ω* while the second one concerns the scattering problem. In other words we consider (15) in whole of *ℝ* ^2^. For a given incident wave *u* ^*i*^, which solves ∇^2^ *u* ^*i*^ = −*k* ^2^ *u* ^*i*^ in *ℝ* ^2^, we take the Sommerfeld condition as the boundary condition which says that the scattered field *u*_*η*_^*s*^ = *u*_*η*_ − *u* ^*i*^ satisfies

(17) $$\begin{array}{c} \partial_{r}u_{\eta }^{s}-iku_{\eta }^{s}=o\left(\;r^{-1/2}\;\right) \end{array}$$

for *r* = |*x* | → *∞*, uniformly in the angle variable.

**Remark 7 .**

Note that for (15) we have not given any boundary conditions; instead we have considered an arbitrary sequence of solutions; however, the uniqueness of solution of the scattering problem will be proven for every *η*. To state the main results, we rewrite (15) as a system:

(18) ∇·jη=−k2uη,jη=aη∇uη.

Comparing with (12), we see that *j*_*η*_ represents (up to a factor and perhaps a rotation) the horizontal electric field ${\vec{E}}_{\eta }$ and since the magnetic field ${\vec{H}}_{\eta }(x)=(0,0,u_{\eta }(x))$, system (18) is nothing but (12) itself.

**Theorem 8 (upscaled equations).**

Let the matallic geometry be given by Σ_*η*_ (Figure 3) on a domain *Ω* ⊂ *ℝ* ^2^ and let the coefficient *a*_*η*_≔*ε*_*η*_^−1^ be as in (16). On *ε*_*r*_ ∈ *ℂ* we assume that either Im⁡(*ε*_*r*_) > 0 or *ε*_*r*_ < 0 = Im⁡(*ε*_*r*_). Let (*u*_*η*_)_*η*>0_ be the sequence of solutions of (15) such that $u_{\eta }\overset{w}{⇀}u$ in *L* ^2^(*Ω*) for *η* → 0. We define *U* ∈ *L* ^2^(*Ω*) as the function

(19) $$\begin{array}{c} U\left(\;x\;\right)≔\left\{\begin{array}{cc} u\left(x\right) & for\;\;x\in \Omega \setminus R,\\ N_{w}^{-1}u\left(x\right) & for\;\;x\in R, \end{array}\right. \end{array}$$

where *N*_*w*_ is defined by (36). Then the function ∇*U* ∈ *L* ^2^(*Ω*). The field *j*_*η*_ = *a*_*η*_∇*u*_*η*_ converges weakly to some *j* in *L* ^2^(*Ω*; *ℂ* ^2^) which is given by

(20) $$\begin{array}{c} j=\left\{\begin{array}{cc} \nabla U & in\;\;\Omega \setminus \bar{R},\\ \alpha \nabla U & in\;\;R. \end{array}\right. \end{array}$$

Moreover, the limit functions satisfy the system

(21) $$\begin{array}{c} \nabla \cdot \left(\;a_{eff}\nabla U\;\right)=-k^{2}\mu_{eff}U\;in\;\;\Omega , \end{array}$$

where

(22) aeff≔α00α,μeff=Nwfor  x∈R,aeff≔1001,μeff=1for  x∈Ω∖R¯.

By applying Theorem 8 for *Ω*≔*B*_*r*_0__(0) with a large radius *r*_0_ > 0, we can treat the scattering problem with an incoming wave generated at infinity. We obtain the strong convergence of the scattered field outside the metallic obstacle and we identify the limit *U*(*x*) as the solution of the effective diffraction problem. We define the exterior domain outside of *R* as $R^{ext}≔{\mathbb{R}}^{2}\setminus \bar{R}$.

**Theorem 9 (effective scattering problem).**

Let the metallic gratings be given by Σ_*η*_ (Figure 1) and the coefficient *a*_*η*_(*x*)≔*ε*_*η*_^−1^(*x*) be as in (16). Assume further that *u* ^*i*^ is an incident wave solving the free space equation ∇^2^ *u* ^*i*^ = −*k* ^2^ *u* ^*i*^ on *ℝ* ^2^ and *u*_*η*_ is the unique sequence of solutions to (15) such that *u*_*η*_^*s*^ = (*u*_*η*_ − *u* ^*i*^) satisfies (17) and that the solution sequence satisfies the uniform bound

(23) $$\begin{array}{c} \overset{}{\underset{R}{\int }}{\left|\;u_{\eta }\;\right|}^{2}\leq C. \end{array}$$

Then *u*_*η*_ → *U*  strongly in *L*_*loc*_^2^(*R* ^*ext*^) with uniform convergence for all derivatives on any compact subset of *R* ^*ext*^. The effective field *U* : *ℝ* ^2^ → *ℂ* is determined as the unique solution of the upscaled equation

(24) $$\begin{array}{c} \nabla \cdot \left(\;a_{eff}\nabla U\;\right)=-k^{2}\mu_{eff}U\;in\;\;R^{2} \end{array}$$

with (17) for the scattered field (*U* − *u* ^*i*^).

#### 1.5.1. Interface Conditions

The homogenized equation (24) should be understood in the sense of distributions on the whole of *ℝ* ^2^. The exterior field $U\in H^{1}(B_{r}(0)\setminus \bar{R})$ for every large radius *r*; hence its trace on ∂*R* from outside, denoted by *U* ^+^, is a well-defined element of *H* ^1/2^(∂*R*). Note that as ∇*U* belongs to *L* ^2^(*B*_*r*_), the function *U*(·, ·) is an element of *H*_loc_^1^(*ℝ*). This helps us to define traces of *U* on the horizontal boundary parts from the inside. Moreover, we have the information that the distributional divergence of the vector field *j* = *a*_eff_∇*U* is of class *L*_loc_^2^(*ℝ* ^2^).

We define the transmission condition on the boundary ∂*R* of *R* with using traces from inside and outside of *R*. We denote by superscript + (resp., by −) traces from outside (resp., by inside); then problem (24) can be rewritten as

(25) ∇2U+k2U=0in  Rext,∇·aeff∇U+Nwk2U=0in  R

with the transmission (interface) conditions

(26) U+=U−on  ∂R,∇U+·n→=aeff∇U−·n→on  ∂R,

where *N*_*w*_ is defined in (36).

## 2. Derivation of the Effective Model

### 2.1. A Priori Estimates

**Lemma 10 .**

For an *ε*_*r*_ ∈ *ℂ* with Im(*ε*_*r*_) > 0, let *a*_*η*_ be defined as in (16). Then there exists a *λ* ∈ *ℂ* such that

(27) Im⁡λaη≥C0aη,

where *C*_0_ > 0 is independent of *η*.

**Proof**

Let *x* ∈ Σ_*η*_ (if *ε*_*r*_ = *a* + *ib* with *b* > 0, then *a*_*η*_ = *η* ^2^((*a* − *ib*)/(*a* ^2^ + *b* ^2^))). For an arbitrary small *δ* > 0, let us define *λ*≔−1 + *δi* such that *δ*|Re(*a*_*η*_)| ≤ −(1/2)Im⁡(*λa*_*η*_). There exists a constant *C*_0_ > 0 such that

(28) Im⁡λaη−Im⁡aη+δRe⁡aη≥−12Im⁡aη≥C0aη.

For *x* ∈ *Ω*∖Σ_*η*_, we have

(29) Im⁡λ·1=δ≥C0,where  C0=δ.

**Lemma 11 (gradient estimate).**

Suppose that the solution (*u*_*η*_)_*η*>0_ of (15) is a bounded sequence in *L* ^2^(*Ω*); that is, sup_*η*>0_⁡‖*u*_*η*_‖_*L*^2^(*Ω*)_ < *∞*. Then for every compactly contained subdomain *Ω*′ ⊂ ⊂*Ω*, the following estimate holds:

(30) $$\begin{array}{c} \overset{}{\underset{\Omega^{\prime }}{\int }}\left|\;a_{\eta }\;\right|{\left|\;\nabla_{x}u_{\eta }\;\right|}^{2}dx\leq C, \end{array}$$

where *C* is independent of the scale parameter *η*.

**Proof**

Since $\bar{R}\subset \Omega$, there exists a subdomain *Ω*′ ⊂ ⊂*Ω* such that $\bar{R}\subset \Omega^{\prime }\subset \subset \Omega$. Without loss of generality, let us assume that Σ_*η*_ ⊂ *Ω*′ and take a* cut-off* function Θ ∈ *C*_0_^*∞*^(*Ω*; [0,1]), where Θ(*x*) = 1 on *Ω*′. We test (15) with $\Theta^{2}(\cdot ){\bar{u}}_{\eta }(\cdot )$, where ${\bar{u}}_{\eta }(\cdot )$ is the complex conjugate of *u*_*η*_(·). This gives

(31) ∫Ωaη∇xuη2Θ2 dx=∫Ωk2uη2Θ2 dx−2∫Ω∖Ω′aη∇xuη·∇xΘΘu¯η dx.

We employ Lemma 10. For a *λ* ∈ *ℂ*, we multiply (31) by *λ* and equate its imaginary part and rearrange the factors of the second integrand which will yield

(32) C0∫Ωaη∇xuη2Θ2 dx≤λ∫Ωk2uη2Θ2 dx+2λ∫Ω∖Ω′√aη∇xuηΘ·√aηuη∇xΘdx⟹C0∫Ωaη∇xuη2Θ2 dx≤λ∫Ωk2uη2Θ2 dx+2λC04λ∫Ωaη∇xuη2Θ2 dx+λC0∫Ω∖Ω′aηuη2∇xΘ2dx⟹C02∫Ωaη∇xuη2Θ2 dx≤λ∫Ωk2uη2Θ2 dx+2λ2C0∫Ω∖Ω′aη∇xΘ2uη2dx,

where in the second step we used* Young's inequality*. We see that the first integral on the r.h.s. of (32) is bounded by the *L* ^2^-boundedness assumption on *u*_*η*_ whereas the second integral on the r.h.s. is bounded by the boundedness of |*a*_*η*_| and ‖*u*_*η*_‖_*L*^2^(*Ω*)_ < *∞*. Using the fact that Θ(*x*) = 1 on *Ω*′, we have ∫_*Ω*′_|*a*_*η*_||∇_*x*_ *u*_*η*_|^2^ *dx* ≤ ∫_*Ω*_|*a*_*η*_||∇_*x*_ *u*_*η*_|^2^Θ^2^ *dx* ≤ *C*, where *C* is independent of *η* and *u*_*η*_.

### 2.2. An Eigenvalue Problem in the Unit Cell *Y*

Let us consider the eigenvalues 0 < *λ*_1_ ≤ *λ*_2_ ≤ ⋯≤*λ*_*n*_ ≤ ⋯ of the problem

(33) Δζ+λζ=0on  Σ,ζy∂Σ=0

and we denote {*ζ*_*n*_} the associated normalized eigenfunctions in *H*_0_^1^(Σ), so that {*ζ*_*n*_} is an orthonormal basis of *L* ^2^(Σ). Since *ε*_*r*_ ∈ *ℂ* with Im(*ε*_*r*_) > 0, *k* ^2^ *ε*_*r*_ satisfies the condition

(34) $$\begin{array}{c} k^{2}\varepsilon_{r}\notin \left\{\;\lambda_{n}:n\in N\;\right\}. \end{array}$$

We set

(35) wy∑n∈Nk2εrλn−k2εr∫Σζnydyζny,

(36) Nw1+∫Σwydy=1+∑n∈Nk2εrλn−k2εr∫Σζnydy2.

Let us consider the following boundary value problem:

(37) Δw+k2εrw=−k2εrin  Σ,wy=0on  ∂Σ.

By [23, theorem  8.22], it follows that (i) *M*_*w*_(*y*) is a solution of (37) and this solution is unique if *k* ^2^ *ε*_*r*_ ≠ *λ*_*n*_ for all *n* and (ii) if condition (34) is not fulfilled then (37) has no solution.

In the next theorem we will analyze the behavior of *u*_*η*_ as *η* → 0 in the sense of two-scale convergence, compare [15]. We notice that the geometry is not only periodic in the *x*_1_-direction but it is also periodic with respect to to the cell *Y*≔(−1/2, 1/2)×(−1/2, 1/2). The metal part in the cell *Y* is given by Σ ⊂ *Y*; see Figure 2.

We recall that the sequnce (*u*_*η*_)_*η*>0_ is weakly convergent to *u* ∈ *L* ^2^(*Ω*). We define a function *u*_0_(*x*, *y*)≔*u*_0_(*x*_1_, *x*_2_, *y*_1_, *y*_2_) as

(38) u0x,y=ux∀x∉R,  ∀y∈Y,Nw−1ux1+wy∀x∈R,  ∀y∈Σ,Nw−1ux∀x∈R,  ∀y∈Y∖Σ,

where *w*(*y*) is a *Y*-periodic function defined in (35). We have defined *u*_0_ in such a way that, for every *x* ∈ *Ω*, there holds *u*(*x*) = ∫_*Y*_ *u*_0_(*x*, *y*)*dy*. We will show in next theorem that $u_{\eta }\overset{2}{⇀}u$ as *η* → 0.

**Lemma 12 (two-scale limit).**

Let (*u*_*η*_)_*η*>0_, weakly converging to *u* in *L* ^2^(*Ω*), be a sequence of solutions of (15). Then for the function *u*_0_ defined in (38) it holds that $u_{\eta }\overset{2}{⇀}u_{0}$.

Outside of *R*, the strong convergence *u*_*η*_ → *u* holds in *L* ^2^(*Ω*∖*R*). More precisely, *u*_*η*_ together with all its derivatives converges uniformly on every compact subset $\Omega^{\prime }\subset \subset \Omega \setminus \bar{R}$.

**Proof**

We divide the proof into three steps.

(i) From the assumption on *u*_*η*_ and the estimate (30), the sequences (*u*_*η*_)_*η*>0_ and (*η*∇*u*_*η*_)_*η*>0_ are bounded in *L* ^2^(*Ω*). Then there exists *u*_0_ : *Ω* × *Y* → *ℂ* ^2^ such that, up to a subsequence, $u_{\eta }\overset{2}{⇀}u_{0}$ and $\eta \nabla u_{\eta }\overset{2}{⇀}\nabla_{y}u_{0}$ as *η* → 0. As a *Y*-periodic function, *u*_0_ and ∇_*y*_ *u*_0_ can be extended by periodicity to all *y* ∈ *ℝ* ^2^. This shows that ∇_*y*_ *u*_0_ ∈ *L*_loc_^2^(*ℝ* ^2^) which implies that *u*_0_(*x*, ·) belongs to *H*_loc_^1^(*ℝ* ^2^), in particular, in *H* ^1^(Σ) and has a trace on ∂Σ. In other words, $u_{0}(x,\cdot )\in H^{1}(\Sigma )\cap C(\bar{\Sigma })$ does not jump accross ∂Σ by trace theorem (cf. [19, theorem  5.5.1]).

Next, we investigate the coefficient *a*_*η*_ = 1 on the set *Ω*∖Σ_*η*_. From (30), it follows that sup_*η*>0_⁡‖∇*u*_*η*_‖_*L*^2^(*Ω*∖Σ_*η*_)_ ≤ *C* < *∞* which implies *η*∇*u*_*η*_  1_*Ω*∖Σ_*η*__ → 0 strongly in *L* ^2^(*Ω*). Since strong convergence implies the two-scale convergence, by localisation Lemma (cf. [3]) the two-scale limit *χ*_0_ vanishes a.e. in *R* × (*Y*∖Σ) and in (*Ω*∖*R*) × *Y*. Due to ∇_*y*_ *u*_0_ = *χ*_0_, it implies that the function *u*_0_(*x*, ·) is constant in *Y*∖Σ and for *x* ∈ *R*; and it is constant everywhere for *x* ∉ *R*. We use this *y*-independence to define a function *U* ∈ *L* ^2^(*Ω*) as

(39) u0x,y≔Uxforx,y∈Ω∖R×Y∪R×Y∖Σ.

We note that, at this stage of the proof, *u*_0_ and *U* are defined as the two-scale limit of *u*_*η*_ and by (39), respectively.

(ii)* Characterisation of Two-Scale Limit for x* ∈ *R*. We claim that, for a.e. *x* ∈ *R*, the function *u*_0_(*x*, ·), which belongs to *H* ^1^(Σ), solves the linear boundary value problem

(40a) Δyu0+k2εru0=0in  Σ,

(40b) u0x,y=Uxon  ∂Σ,

(40c) u0·,−12=u0·,12,

where (40a), (40b), and (40c) hold in the distributional sense in Σ. To verify this, we choose *φ*(*x*, *y*) = Θ(*x*)*ψ*(*y*), where Θ ∈ *C*_0_^*∞*^(*R*; [0,1]) and *ψ* ∈ *C* ^*∞*^(*Y*; [0,1]) a periodic function on Σ with supp⁡(*ψ*)∩(*Y*∖Σ) = *ϕ*. Using *φ*_*η*_(*x*) = *φ*(*x*, *x*/*η*) as the test function in (15), we obtain

(41) ∫R∇·aη∇uηΘxψxηdx+k2∫RuηΘx·ψxηdx=0⟹−∫Raη∇uη∇Θxψxη+1η∇ψxηΘxdx+k2∫RuηΘxψxηdx=0.

Passing the two-scale limit as *η* → 0

(42) ∫R∇·aη∇uηΘxψxηdx+k2∫RuηΘxψxηdx=0⟹−εr−1∫R∫Y∇yu0x,y∇yψyΘxdx dy+k2∫R∫Yu0x,yψyΘxdx dy=0⟹∫R∫YΔyu0x,y+k2εru0x,yψyΘxdx dy=0.

Since Θ was chosen arbitrarily, (40a), (40b), and (40c) hold. For every *x* ∈ *R*, we write *u*_0_(*x*, ·) = *U*(*x*)(1 + *w*(·)), where *w* ∈ *H*_0_^1^(Σ) and *w*(·) is *Y*-periodic. Clearly, *w* ∈ *H*_0_^1^(Σ) satisfies the equation

(43a) $$\begin{array}{c} \Delta_{y}w+k^{2}\varepsilon_{r}={-k}^{2}\varepsilon_{r}\;in\;\;\Sigma , \end{array}$$

(43b) $$\begin{array}{c} w\left(\;y\;\right)=0\;on\;\;\partial \Sigma , \end{array}$$

(43c) $$\begin{array}{c} w\left(\;\cdot \;\right)is\;\;Y\text{-}periodic. \end{array}$$

Then, for *k* ^2^ *ε*_*r*_ ≠ *λ*_*n*_, as shown in Section 2.2, we express *w* uniquely in terms of the orthonormal basis {*ζ*_*n*_}. Note that if the condition (34) is violated, the equation in *w* has no solution and we are led to *u*_0_(*x*, *y*) = *U*(*x*) = 0.

Therefore, to sum up, we obtain the two-scale limit as *u*_0_(*x*, *y*)≔*U*(*x*)(1 + ∑_*n*∈*ℕ*_(*k* ^2^ *ε*_*r*_/(*λ*_*n*_ − *k* ^2^ *ε*_*r*_))

*ζ*_*n*_(*y*)∫_Σ_ *ζ*_*n*_(*y*)*dy*), provided (34) holds. Consequently, for *x* ∈ *R*, the weak limit *u* satisfies

(44) ux∫Yu0x,ydy=Ux∫Y1+wydy=UxNw⟹UxuxNw−1.

Therefore the two-scale limit is given by

(45) u0x,y=ux∀x∉R,  ∀y∈Y,Nw−1ux1+wy∀x∈R,  ∀y∈Σ,Nw−1ux∀x∈R,  ∀y∈Y∖Σ,

where *w*(*y*) = ∑_*n*∈*ℕ*_(*k* ^2^ *ε*_*r*_/(*λ*_*n*_ − *k* ^2^ *ε*_*r*_))*ζ*_*n*_(*y*)∫_Σ_ *ζ*_*n*_(*y*)*dy*.

(iii)* Strong Convergence Outside of R*. We know that *u*_0_(*x*, *y*) = *u*(*x*) = *U*(*x*) holds for a.e. *x* ∈ *Ω*∖*R* and for all *y* ∈ *Y*. Moreover, by the assumption on *u*_*η*_ and estimate (30), we have ‖*u*_*η*_‖_*L*^2^(*Ω*∖*R*)_ + ‖∇*u*_*η*_‖_*L*^2^(*Ω*∖*R*)_ ≤ *C* < *∞*. This then implies that *u*_*η*_, up to a subsequence, is strongly convergent to *U* in *L* ^2^(*Ω*∖*R*) by* Aubin-Lion's Lemma*, compare [24]. The uniform convergence on compact subsets of *u*_*η*_ and of all its derivatives is a consequence of the fact that *u*_*η*_ Helmholtz equation Δ*u*_*η*_ + *k* ^2^ *u*_*η*_ = 0.

With the help of Lemma 12, we can completely determine the two-scale limit of the sequence (*u*_*η*_)_*η*>0_ if we know the function *U*(*x*) which is defined in (39). Now we collect the properties of ${\vec{j}}_{\eta }$, its weak limit $\vec{j}$, and its two-scale limit ${\vec{j}}_{0}$.

**Proposition 13 .**

Let $u_{\eta }\overset{w}{⇀}u$ be as in Lemma 12 and *U* be given by (39). For ${\vec{j}}_{\eta }=a_{\eta }\nabla u_{\eta }$, we suppose that ${\vec{j}}_{\eta }\overset{w}{⇀}\vec{j}≔(j_{1},j_{2})$ in *L* ^2^(*Ω* : *ℝ* ^2^). Then $\vec{j}$ is characterized as follows:

(i) The sequence ${\vec{j}}_{\eta }$ converges in the sense of two scales to ${\vec{j}}_{0}$ which is given by
  (46) $$\begin{array}{c} {\vec{j}}_{0}\left(\;x,y\;\right)=\left\{\begin{array}{cc} 0 & for\;\;x\in R,\;\;y\in \Sigma ,\\ \alpha^{-1}\vec{j}\left(x\right)\chi \left(y\right) & for\;\;x\in R,\;\;y\in Y\setminus \Sigma ,\\ \vec{j}\left(x\right) & for\;\;x\in \Omega \setminus \bar{R},\;\;y\in Y. \end{array}\right. \end{array}$$
(ii) The limit ∇*U* ∈ *L* ^2^(*Ω*) and it holds:
  (47) $$\begin{array}{c} \vec{j}\left(\;x\;\right)=\left\{\begin{array}{cc} 0 & for\;\;x\in R,\;\;y\in \Sigma \\ \alpha \nabla U\left(x\right) & for\;\;x\in R,\;\;y\in Y\setminus \Sigma ,\\ \nabla U\left(x\right) & for\;\;x\in \Omega \setminus \bar{R},\;\;y\in Y. \end{array}\right. \end{array}$$

**Remark 14 .**

We would like to point out a major difference in our *j*_0_ and the limit function *j*_0_ in [3]. In [3], the authors worked with a rectangular metallic subpart Σ of type (−*γ*, *γ*)×(−1/2, 1/2) and therefore by defining a suitable test function, they have shown that the first component of *j*_0_ vanishes and they obtain *j*_0_ = (0, *α* ^−1^∂_*x*_2__ *U*)  for  *x* ∈ *R*  and  *y* ∈ *Y*∖Σ. This is clearly not the case in this paper as the metallic subpart Σ is chosen to be sinusoidal along *y*_2_-axis due to geometry of the metallic structure given by Figure 1. This will lead to nonvanishing componenets of *j*_0_ and we end up having a different *j*_0_ compared to that in [3] and hence, we will obtain a different upscaled equation.

**Proof**

By (30), it follows that ${\vec{j}}_{\eta }$ is bounded in *L*_loc_^2^(*Ω*) which implies that up to a subsequence ${\vec{j}}_{\eta }$ two-scale converges to some ${\vec{j}}_{0}$. The weak limit would then be given as $\vec{j}(x)=\int_{Y}{\vec{j}}_{0}(x,y)dy$.

*The Field outside of R*. For $x\in \Omega \setminus \bar{R}$, ${\vec{j}}_{\eta }=\nabla u_{\eta }$. Then by Lemma 12, ∇*u*_*η*_ → ∇*U* uniformly on compact subsets of $\Omega \setminus \bar{R}$. This leads to

(48) j→0x,·=j→x=∇Uxfor  a.e.x,y∈Ω∖R¯×Y.

*The Field in the Metal Part of R*. We note that |*a*_*η*_| ≤ *Cη* ^2^ in Σ_*η*_ and |*a*_*η*_| ≤ *C* in *R*∖Σ_*η*_; therefore (30) gives ‖*j*_*η*_‖_*L*^2^(Σ_*η*_)_^2^ ≤ *Cη* ^2^ and ‖*j*_*η*_‖_*L*^2^(*R*∖Σ_*η*_)_^2^ ≤ *C*. This implies ${\overset{-}{\lim}}_{\eta \to 0}{\left\|j_{\eta }\right\|}_{L^{2}(\Sigma_{\eta })}^{2}=0$ and by [21, theorem  17], we have ${\vec{j}}_{0}(x,y)=0$ a.e. in *R* × Σ. Moreover, *j*_0_(*x*, *y*) = *j*_0_(*x*) = ∇*U*(*x*) for a.e. (*x*, *y*) ∈ *R* × (*Y* − Σ).

*Divergence of j*_0_. Due to boundedness assumption on *u*_*η*_, by (18) we have ‖∇·*j*_*η*_‖_*L*^2^(*Ω*)_ ≤ *k* ^2^  sup_*η*>0_⁡‖*u*_*η*_‖_*L*^2^(*Ω*)_ < *∞*, ∀*η* > 0. For Θ ∈ *C*_0_^*∞*^(*Ω*) and *ψ* ∈ *C*_per_^*∞*^(*Y*), we test (18) by Θ(*x*)*ψ*(*x*/*η*) which gives

(49) 0=limη→0⁡∫Ωη∇·jηΘxψxηdx=−limη→0⁡∫Ωηjη·∇Θxψxη+1ηΘx∇x/ηψxηdx=−∫Ω∫Yj0x,y·∇yψyΘxdx dy.

Since Θ ∈ *C*_0_^*∞*^(*Ω*) is arbitrary, ∫_*Y*_∇_*y*_ *ψ*(*y*) · *j*_0_(*x*, *y*)  *dy* = 0 for a.e. *x* ∈ *Ω* which implies ∇_*y*_ · *j*_0_(*x*, ·) = 0 for *y* ∈ *Y* in distributional sense. This shows that ${\vec{j}}_{0}(x,y)$ is independent of *y*; that is, ${\vec{j}}_{0}(x,y)=\vec{j}(x)$, some function in *x* only.

Next we determine the relation between ${\vec{j}}_{0}$ and *U* as shown in [3]. We define Ξ≔{*ψ* ∈ *L* ^2^(*ℝ* ^2^; *ℂ* ^2^):∇·*ψ* = 0, *ψ* is* Y*-periodic, and *ψ* = 0  in  Σ}. We choose a test function *φ*(*x*, *x*/*η*)≔Θ(*x*)*ψ*(*x*/*η*), where Θ ∈ *C*_0_^*∞*^(*R*; [0,1]) and *ψ* ∈ Ξ. We use ${\vec{j}}_{\eta }{|}_{\Omega \setminus \Sigma_{\eta }}=\nabla u_{\eta }$ and ∇·*φ*(*x*, *x*/*η*)≔∇·(Θ(*x*)*ψ*(*x*/*η*)) = *ψ*(*x*/*η*)·∇Θ(*x*) as ∇_*y*_ · *ψ*(*y*) = 0. Then

(50) ∫Y∫Ωj→0x,y·ψyΘxdx dy=limη→0⁡∫Ωj→ηx·ψxηΘxdx=limη→0⁡∫Ω∇uηx·ψxηΘxdx=−limη→0⁡∫Ωuηxψxη·∇Θxdx=−∫Y∫Ωu0x,yψy·∇Θxdx dy.

Since *ψ*(*y*)|_Σ_ = 0 and is nonvanishing in *Y*∖Σ and by (39) it implies that *u*_0_(*x*, *y*) = *U*(*x*), all these lead to

(51) ∫Y∫Ωj→0x,y·ψyΘxdx dy=−∫Y∫ΩUxψy·∇Θxdx dy⟹∫Y∫Ωj→0x,y·ψyΘxdx dy=−∫ΩUx∇Θxdx·∫Yψydy.

Therefore, *j*_0_(*x*, *y*) = ∇*U*(*x*) for $(x,y)\in R\times (Y\setminus \Sigma )\cup (\Omega \setminus \bar{R})\times Y$ and for reminder *j*_0_(*x*, *y*) = 0 for (*x*, *y*) ∈ *R* × Σ.

*Proof of (i)*. To conclude this part, the arguments rely on that of [3]. We consider ${\vec{j}}_{1}(x,y)≔\alpha^{-1}\vec{j}(x)\chi (y){|}_{Y\setminus \Sigma }$. We intend to show that ${\vec{j}}_{1}(x,\cdot )={\vec{j}}_{0}(x,\cdot )$ for almost every *x* ∈ *R*. To show this, we define a function ${\vec{\psi }}_{0}(y)={\vec{j}}_{0}(x,y)-{\vec{j}}_{1}(x,y)$. We notice that (i) ∫_*Y*_ *j*_0_(*x*, *y*)*dy* = ∫_*Y*_ *j*(*x*)*dy* = *j*(*x*) and $\int_{Y}j_{1}(x,y)dy=\int_{Y}{\left.\alpha^{-1}\vec{j}(x)\chi (y)\right|}_{Y\setminus \Sigma }dy=j\left(x\right)\alpha^{-1}\left|Y\setminus \Sigma \right|=j(x)$, (ii) ∇_*y*_ · *j*_0_(·, *y*) = 0 and ∇_*y*_ · *j*_1_(·, *y*) = 0, and (iii) ${\left.j_{0}(x,y)\right|}_{\Sigma }={\left.\left[\alpha^{-1}\vec{j}\left(x\right){\left.\chi \right|}_{Y\setminus \Sigma }\right]\right|}_{\Sigma }=0$. This implies that (51) holds good for ${\vec{\psi }}_{0}$ as well as for the conjugate $\bar{{\vec{\psi }}_{0}}$ of ${\vec{\psi }}_{0}$. Therefore using the fact that $\int_{Y}\bar{{\vec{\psi }}_{0}}dy=0$ from (51), we have

(52a) ∫Y∫Ωj→0x,y·ψ→0¯Θxdx dy=0,

(52b) ∫Y∫Ωj→1x,y·ψ→0¯Θxdx dy=∫Y∫Ωα−1j→xχyY∖Σ·j→0x,y−j→1x,yΘxdx dy=∫Ωα−1jx·∫Yj0x,ydy−α−2jx2·∫Y∖Σχ2yY∖Σ dyΘxdx=∫Ωα−1jx2−α−1jx2Θxdx=0

Substraction of (52a) and (52b) gives $\int_{\Omega }\int_{Y}\psi_{0}(y)\cdot \bar{{\vec{\psi }}_{0}}(y)\Theta (x)dx\;dy=\int_{\Omega }\int_{Y}{\left|\psi_{0}\left(y\right)\right|}^{2}\Theta \left(x\right)dx\;dy=0$. Since Θ is arbitrary, therefore *ψ*_0_(*y*) = 0, which shows that ${\vec{j}}_{0}(x,\cdot )={\vec{j}}_{1}(x,\cdot )=\alpha^{-1}\vec{j}(x)\chi (y){|}_{Y\setminus \Sigma }$. This completes the proof of part (i).

*Proof of (ii)*. To verify the claim, let us choose $\psi (y)={\left.\left({\hat{e}}_{1},{\hat{e}}_{2}\right)\chi \left(y\right)\right|}_{Y\setminus \Sigma }$ and Θ ∈ *C*_0_^*∞*^(*Ω*). Note that $\int_{Y}\psi (y)dy=\alpha ({\hat{e}}_{1},{\hat{e}}_{2})=\left(1-\left(3/2\right)\gamma \right)({\hat{e}}_{1},{\hat{e}}_{2})$. Then from (51), we have

(53) −α∫ΩUxe^1,e^2·∇Θxdx dy=∫Y∫Ωj→0x,y·ψyΘxdx=∫Y∫Ωj→0x,y·χyY∖ΣΘxdx dy=∫Ω∖Rαe^1,e^2·j→xΘxdx+∫Re^1,e^2·j→xΘxdx.

It follows that ∇*U* ∈ *L* ^2^(*Ω*) and we find also that $\vec{j}(x)=\alpha \nabla U(x)$ for *x* ∈ *R* and $\vec{j}(x)=\nabla U(x)$ for *x* ∈ *Ω*∖*R*; that is, $\vec{j}(x)=a(x)\;\nabla U(x)$, where *a*(*x*) = *α* for *x* ∈ *R* and *a*(*x*) = 1 for *x* ∈ *Ω*∖*R*.

## 3. Proofs of Theorems 8 and 9

**Proof of Theorem 8.**

The proof of Theorem 8 is a straightforward consequence of Lemmas 11 and 12 and Proposition 13. It is being shown that if, for any subdomain *Ω*′ with *R* ⊂ *Ω*′ ⊂ ⊂*Ω*, ${\vec{j}}_{\eta }=a_{\eta }\nabla u_{\eta }$ is bounded in *L* ^2^(*Ω*′), then, up to a subsequence, ${\vec{j}}_{\eta }$ is weakly convergent to some $\vec{j}$ in *L* ^2^(*Ω*′).

By Proposition 13, we have the relation between *U* and $\vec{j}$; that is, the weak and the two-scale limits of ${\vec{j}}_{\eta }$ are given in terms of *U*; see (46) and (47). Since *Ω*′ is arbitrary, the results of Proposition 13 hold good in all *Ω*. Now we obatin the limit problem by dividing the proof into two following cases.

*Case 1*. Let $x\in \Omega \setminus \bar{R}$; then for *ϕ* ∈ *C*_0_^*∞*^(*Ω*) from (15) we have

(54) −∫Ω∖R¯aη∇uηx·∇ϕxdx=−k2∫Ω∖R¯uηϕ dx⟹η→0−∫Ω∖R¯∫Y∇Ux·∇ϕxdx dy=−k2∫Ω∖R¯∫YUxϕxdx dy.

*Case 2*. Let *x* ∈ *R*; then again for *ϕ* ∈ *C*_0_^*∞*^(*Ω*) from (15) we have

(55) −∫Raη∇uηx·∇ϕxdx=−k2∫Ruηϕ dx⟹η→0−∫R∫Yj0x,y·∇ϕxdx dy=−k2∫R∫Yu0x,yϕxdx dy⟹η→0−∫R∫Y∖Σα−1jxχy·∇ϕxdx dy=−k2∫R∫Yu0x,yϕxdx dy⟹η→0−α∫R∫Y∇Ux·∇ϕxdx dy=−k2Nw∫RUxϕxdx.

The combination of (54) and (55) gives the limit problem as

(56) $$\begin{array}{c} \nabla \cdot \left(\;a_{eff}\nabla U\;\right)=-k^{2}\mu_{eff}U\;in\;\;\Omega , \end{array}$$

where

(57) aeff≔α00α,μeff=Nwfor  x∈R,aeff≔1001,μeff=1for  x∈Ω∖R¯.

Here we can compare our upscaled equation with the limit problem obtained in [3], especially for *x* ∈ *R*. Due to their rectangular metallic gratings inside* R*, the component along *x*_1_ direction vanishes; that is, the first component of $\vec{j}=0$ and thus the authors obtained their upscaled equation as *α*∂_*x*_2__^2^ *U* = −*k* ^2^ *μ*_eff_ *U*.

**Proof of Theorem 9.**

The proof is devided into three steps which are demonstrated below.

*(i) Uniqueness of the Limit Problem*. With a fixed incident field *u* ^*i*^ we will show that the limit problem (24) has a unique solution. On the contrary, let us assume that *U*_1_ and *U*_2_ are the two solutions of (24) and set *u* = *U*_1_ − *U*_2_. We consider the equations satisfied by difference of two solutions as

(58) ∇·aeff∇u=−k2μeffuin  R2,

(59) ∂ru−iku=or−1/2for  r⟶∞.

We claim that *u*(*x*, *y*) = 0  for  *x* ∈ *ℝ*  and  *y* ∈ *Y*. The main ingredient for this uniqueness result is Rellich's first lemma and the fact that *a*_eff_ is real and *μ*_eff_ has positive imaginary part. In fact *a*_eff_ is identity and *μ*_eff_ is 1 outside of *R*. Let us denote the surface of a sphere *B*_*r*_(0) of radius *r* by *S*_*r*_  (≔∂*B*_*r*_(0)), where *r* is chosen so large such that *R* ⊂ *B*_*r*_(0). Let *r*_0_ be such *r*; then by (59), we have

(60) limr→+∞⁡∫Sr0∂ru2−k2u2︸real+2kIm⁡u∂ru¯=limr→+∞⁡∫Sr0∂ru−iku2=0.

This gives

(61) $$\begin{array}{c} \overset{}{\underset{S_{r_{0}}}{\int }}Im\left(\;u\partial_{r}\bar{u}\;\right)ds\leq 0. \end{array}$$

Now we multiply (58) by $\bar{u}$ and integrate over *B*_*r*_0__(0). Since (58) holds only in the sense of distributions and due to possible jumps on Γ_hor_, we approximate $\bar{u}$ by smooth functions. By divergence theorem we have

(62) ∫Br0∇·uaeff∇u¯dv=∫Sr0aeffu∇u¯·n→ ds⟹∫Rα∇u2+∫Br00∖R∇u2−∫Br00k2μeffu2=∫Sr0∂ruu¯=−∫Sr0u∂ru¯.

The surface integral on r.h.s. of (62) is well defined. This can be argued as follows: outside of *R*, *u* is a solution of the Helmholtz equation ∇^2^ *u* = −*k* ^2^ *u* and so it is analytic in the exterior of *R*. Therefore the traces of *u* and ∂_*r*_ *u* are well defined in *S*_*r*_0__, compare [25]. Comparing the imaginary parts of (62) and investing the knowledge of Im(*μ*_eff_) > 0, then

(63) k2Im⁡μeff∫Br00u2=∫Sr0u∂ru¯≤0.

Therefore from (63) we have *u* = 0 in *R*. Since *r*_0_ is chosen arbitrarly, for every *r* from (60) it follows that

(64) $$\begin{array}{c} \overset{}{\underset{S_{r}}{\int }}{\left|\;u\;\right|}^{2}ds⟶0\;as\;\;r⟶+\infty . \end{array}$$

Thus by Rellich's first lemma (which states that the solutions* u* of the Helmholtz equation on an exterior domain satisfying property (64) vanish) we obtain *u* = 0 in all of *ℝ* ^2^ which concludes the proof of the uniqueness property, compare [25].

*(ii) Convergence to the Limit Problem Assuming an L*_*loc*_^2^ *-Bound*. Let the radius *r*_0_ > 0 be such that $\bar{R}\subset B_{r_{0}}(0)$ and set *Ω*≔*B*_*r*_0__(0). We begin with the assumption that

(65) $$\begin{array}{c} t_{\eta }≔{\left(\;\int_{\Omega }^{}{\left|\;u_{\eta }\;\right|}^{2}\;\right)}^{1/2}\leq C\;\forall \eta >0. \end{array}$$

The proof basically follows as the one for Theorem 8. Using (65), up to a subsequence, passing the limit as *η* → 0, we obtain that *U*(*x*) = *u*(*x*)*χ*(*x*)|_*Ω*∖*R*_ + *N*_*w*_^−1^ *u*(*x*)*χ*(*x*)|_*R*_ solves (24).

We only need to verify the radiation condition (17). By Lemma 12 it follows that *u*_*η*_ and ∇*u*_*η*_ are uniformly convergent on every compact subset of *ℝ* ^2^∖*R*. Let us choose *r* < *r*_0_ such that *R* ⊂ ⊂*B*_*r*_(0)⊂⊂*Ω*. By [25, theorem  2.4] and end remark of that theorem, we have from the Sommerfeld radiation condition that the scattered field *u*_*η*_^*s*^ = *u*_*η*_ − *u* ^*i*^ coincides on *ℝ* ^2^∖*B*_*r*_(0) with its Helmholtz representation through values and derivatives of *u*_*η*_ − *u* ^*i*^ on ∂*B*_*r*_(0). By the similar representation formula, using the values and derivatives of *U* − *u* ^*i*^ on ∂*B*_*r*_(0), we can extend *U* into all of *ℝ* ^2^ to a solution of the Helmholtz equation ∇^2^ *U* = −*k* ^2^ *U* outside of *R*. Thus this construction of *U* shows that *U* − *u* ^*i*^ satisfies the Sommerfeld radiation condition. The uniform convergence of *u*_*η*_ → *U* and ∇*u*_*η*_ → ∇*U* on ∂*B*_*r*_(0) implies the uniform convergence of *u*_*η*_ and its derivatives on all compact subsets of exterior of *R*. Finally by uniqueness of the limit from part (i), $u_{\eta }\overset{w}{⇀}u$ as *η* → 0 for the whole sequence. This shows that the Sommerfeld radiation condition holds for *r* = |*x* | → *∞*. which establishes (17).

*(iii) Boundedness of t*_*η*_. In the previous step the limit problem is obtained assuming (65) is true. We will prove that (65) holds true by the method of contradiction. We suppose that *t*_*η*_ → *∞*, up to a subsequence, as *η* → 0. Now we consider the normalized sequence

(66) $$\begin{array}{c} v_{\eta }≔\frac{1}{t_{\eta }}u_{\eta }\;such\;\;that\;\;{\left\|v_{\eta }\right\|}_{L^{2}\left(\Omega \right)}=1, \end{array}$$

Due to linearity, *v*_*η*_ solves the original scaterring field problem with incident field *v*_*η*_^*i*^ = *u* ^*i*^/*t*_*η*_ → 0 as *η* → *∞*. Following the proofs of Lemma 12 and parts (i) and (ii), the function *V* = *vχ*(*x*)|_*Ω*∖*R*_ + *N*_*w*_^−1^ *vχ*(*x*)|_*R*_ is the unique solution of (24) and satisfies the Sommerfeld wave condition. By the construction of *v*_*η*_, we obtain *V* = 0 and therefore *v*_*η*_ → 0 weakly in *L* ^2^(*Ω*).

For outside of *R*, the gradient estimate (30) for *v*_*η*_ remains valid and hence, *v*_*η*_|_*Ω*∖*R*_ remains in a bounded subset of *H* ^1^(*Ω*∖*R*). Then by Rellich compactness lemma ∫_*Ω*∖*R*_|*v*_*η*_|^2^ → 0 as *η* → 0. For inside of *R*, we use the estimate (23) on *u*_*η*_ and since *t*_*η*_ → *∞*, ∫_*R*_|*v*_*η*_|^2^ → 0 as *η* → 0. Therefore ∫_*Ω*_|*v*_*η*_|^2^ → 0 as *η* → 0 but this contradicts the fact that ‖*v*_*η*_‖_*L*^2^(*Ω*)_ = 1. Thus *t*_*η*_ has to be bounded.

## 4. Transmission Properties of the Effective Layer

By Theorems 8 and 9 we have obtained the upscaled Helmholtz equation with effective coefficients. In this section we calculate the corresponding effective reflection and transmission properties of the metallic structure.

Let the rectangle *R* be *ℝ* × (−*h*, 0) for *h* > 0. We assume planar front of waves that reaches the metallic slab (−*h* < *x*_2_ < 0) from above (*x*_2_ > 0). The incoming waves would be partially reflected and partially transmitted through the metallic structure. Before we proceed any further we define the following parameters:

 M= amplitude of the incident wave, where *M* = 1
 
*θ*≔ incident angle, where *θ* ∈ (−*π*/2, *π*/2)
 
*T*≔ complex amplitude and phase shift, where *T* ∈ *ℂ*
  
*A*_*i*_, *B*_*i*_≔ complex amplitudes in the structure, where *A*_*i*_, *B*_*i*_ ∈ *ℂ*, *i* = 1,2
 
*R*≔ complex amplitude of the reflected wave, where *R* ∈ *ℂ*

We write the solution *U* of (24) as
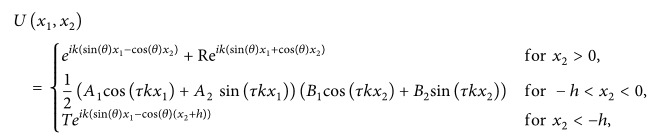
(67)where *τ*≔√(*N*_*w*_/*α*). We are yet to determine the coefficients *A*_*i*_, *B*_*i*_, *R*, and *T* by using the interface (*x*_2_ = 0 and *x*_2_ = −*h*) and transmission conditions (see (26)).

*The Transfer Matrix M*. We will calculate a transfer matrix *M* which basically gives a transformation relation between the solutions on the upper boundary *x*_2_ = 0 and the lower boundary *x*_2_ = −*h*. To be precise, we define a map *M* : *ℂ* ^2^ → *ℂ* ^2^ (i.e., *M* ∈ *ℂ* ^2  ×  2^) as

(68) $$\begin{array}{c} M:\left(\begin{array}{c} U\left(0\right)\\ \nabla U\left(0\right)\cdot \vec{n} \end{array}\right)⟶\left(\begin{array}{c} U\left(-h\right)\\ \nabla U\left(-h\right)\cdot \vec{n} \end{array}\right), \end{array}$$

where $\vec{n}$ is outer normal at *x*_2_ = 0 and *x*_2_ = −*h* which we choose as (0,1) and (0, −1), respectively. In short the matrix *M* maps the vector $\left(U^{+},\nabla U^{+}\cdot \vec{n})=(U(x_{1},0+),\nabla U(x_{1},0+)\cdot \vec{n}\right)$ on the upper boundary to the vector $\left(U(x_{1},-h-),\nabla U(x_{1},-h-)\cdot \vec{n})=(U^{+},\nabla U^{+}\cdot \vec{n}\right)$ on the lower boundary. As the map is seen to be linear, *M* can be expressed as a *ℂ* ^2  ×  2^ matrix. Now we determine the transfer matrix *M* where the two columns are obtained by *M* · (1,0)^*t*^ and *M* · (0,1)^*t*^.

*Columns of M*. To obtain the first column of *M*, we study a solution *U* of the effective system such that *U*(*x*_1_, *x*_2_)|_*x*_2_=0+_ = 1 and $\nabla U\cdot \vec{n}{|}_{x_{2}=0+}=0$. The solution *U* in the interval (−*h*, 0) is given by (67). By transmission conditions we have *U*(*x*_1_, *x*_2_)|_*x*_2_=0+_ = *U*(*x*_1_, *x*_2_)|_*x*_2_=0−_ = 1, *α*∂_*x*_1__ *U*|_*x*_2_=0+_ = *α*∂_*x*_1__ *U*|_*x*_2_=0−_ = 0, and *α*∂_*x*_2__ *U*|_*x*_2_=0+_ = *α*∂_*x*_2__ *U*|_*x*_2_=0−_ = 0. With the help of these conditions, we obtain *A*_2_ = *B*_2_ = 0 and (1/2)*A*_1_ *B*_1_cos(*τkx*_1_) = 1 which gives *U*(*x*_1_, *x*_2_) = cos(*τkx*_2_). With the help of similar transmission condition we obtain

*U*(*x*_1_, *x*_2_)|_*x*_2_=−*h*−_ = *U*(*x*_1_, *x*_2_)|_*x*_2_=−*h*+_ = cos⁡(*τkh*), ∂_*x*_2__ *U*|_*x*_2_=−*h*−_ = *α*∂_*x*_2__ *U*|_*x*_2_=−*h*+_ = *ατk*sin⁡(*τkh*), and ∂_*x*_1__ *U*|_*x*_2_=−*h*−_ = *α*∂_*x*_1__ *U*|_*x*_2_=−*h*+_ = 0. This gives first column of *M* as (cos⁡(*τkh*), *ατk*sin⁡(*τkh*))^*t*^. A similar computation by taking *M* · (0,1)^*t*^ in account will yield the second column of *M* as (−(*ατk*)^−1^sin⁡(*τkh*), cos⁡(*τkh*))^*t*^. Thus the required transfer matrix is given by

(69) $$\begin{array}{c} M≔\left(\begin{array}{cc} \cos\left(\tau kh\right) & -{\left(\alpha \tau k\right)}^{-1}\sin\left(\tau kh\right)\\ -\alpha \tau k\sin\left(\tau kh\right) & -\cos\left(\tau kh\right) \end{array}\right), \end{array}$$

where *τ*≔√(*N*_*w*_/*α*) and *α* = 1 − (3/2)*γ*.

*The Transmission Coefficient*. After having the matrix *M* in hand, our next step is to calculate the transfer coefficient *T*. With the help of matrix *M*, we map the values $\left(U,\nabla U\cdot \vec{n}\right)$ at *x*_2_ = 0+ to the values $\left(U,\nabla U\cdot \vec{n}\right)$ at *x*_2_ = −*h*−; that is, (1 + *R*, *ik*cos⁡(*θ*)(−1 + *R*))*e* ^*ikx*_1_sin⁡(*θ*)^ will get mapped to (*T*, *Tik*cos⁡(*θ*))*e* ^*ikx*_1_sin⁡(*θ*)^. In other words,

(70) M·1+Rikcos⁡θ−1+Reikx1sin⁡θ=T1ikcos⁡θeikx1sin⁡θ.

Here since we are only interested in the transmission coefficient *T*, we eliminate the unknown *R*. Now we follow a simple elimination technique shown in [3] and introduce two vectors *v* ∈ *ℂ* ^2^ and *w* ∈ *ℂ* ^2^ by

(71) vv1v2≔M·1ikcos⁡θ=cos⁡τkh−ikcos⁡θατk−1sin⁡τkh−ατksin⁡τkh−ikcos⁡θcos⁡τkh,w−v2v1=ατksin⁡τkh+ikcos⁡θcos⁡τkhcos⁡τkh−ikcos⁡θατk−1sinτkh.

Since the left hand side of (70) is $R\vec{v}$, multiplying it with *v* ^⊥^ will result in the elimination of *R* from (70). This leads to

(72) $$\begin{array}{c} w\cdot M\left(\begin{array}{c} 1\\ -ik\cos\left(\theta \right) \end{array}\right)=T\;\;w\cdot \left(\begin{array}{c} 1\\ ik\cos\left(\theta \right) \end{array}\right) \end{array}$$

and a straightforward computation yields

(73) T=2ikcos⁡θ2ikcos⁡θcos⁡kτh+sin⁡τkhατk+k2cos2⁡θατk−1,=1cos⁡τkh−i/2sin⁡τkhατ/cos⁡θ+cos⁡θ/ατ=cos⁡τkh−i2sin⁡τkhατcos⁡θ+cos⁡θατ−1.

By (73), we have determined the transmission coefficient *T* which depends on wave number *k*, height of the metallic structure *h*, the aperture volume *α*, effective material parameter *τ*, and the angle *θ*. We note that *τ* = √(*N*_*w*_/*α*), where *N*_*w*_ is defined by the help of an eigenvalue problem in the metallic part Σ and we also notice that *T* depends on the wave number *k* by the relation *N*_*w*_ = *N*_*w*_(*k*). For a rather simple *N*_*w*_, the graph of |*T*|^2^ against the wave number *k* is shown in figure  4 in [3].

Let us focus again on the case of a material that permits perfect plasmon waves, that is, of a lossless material with negative permittivity, *ε*_*r*_ < 0; then *N*_*w*_ ∈ (0,1). Also *α* = 1 − (3/2)*γ* ∈ (0,1), where 0 < *γ* < (1/2). This implies that the term ((*ατ*/cos⁡(*θ*)) + (cos⁡(*θ*)/*ατ*)) in (73) is greater than or equal to 2. Consequently, |*T*| ≤ 1 and we get |*T*| = 1⇔cos⁡(*τkh*) = 1. This corresponds to a resonance of the plasmon waves in the metallic structure (by solving ∇^2^ *U* = −*k* ^2^ *τ* ^2^ *U* for *x*_2_ ∈ (−*h*, 0)) with height *h*.

We see that this effect can also be deduced from the transfer matrix* M* of (68), since for cos⁡(*τkh*) = 1, sin⁡(*τkh*) = 0 and we get the transfer matrix* M = I*, the Identity matrix, corresponding to perfect transmission.
